# Adverse event severity in weekend and after-hours interventional radiology: a multi-institutional quality review

**DOI:** 10.1186/s42155-026-00690-y

**Published:** 2026-05-01

**Authors:** Gavin Wu, Jeffrey Forris Beecham Chick, Adlai Grayson, Arthie Jeyakumar, Andrew G. Kim, David S. Shin, Eric J. Monroe, Mina S. Makary

**Affiliations:** 1 Division of Vascular and Interventional Radiology, Department of Radiology, The Ohio State University Wexner Medical Center, Columbus, OH USA; 2 Division of Vascular and Interventional Radiology, Department of Radiology, University of Southern California, Los Angeles, CA USA; 3 Department of Radiology, University of Washington, Seattle, WA USA; 4 Section of Vascular and Interventional Radiology, Department of Radiology, University of Wisconsin, Madison, WI USA

**Keywords:** Interventional radiology, Adverse events, Procedure timing, After-hours procedures, Weekend procedures, Morbidity and mortality, Quality improvement

## Abstract

**Background:**

Procedures performed after-hours have been associated with worse outcomes in several procedural specialties. Data examining the relationship between procedure timing and adverse event severity in interventional radiology remain limited. This study retrospectively evaluated adverse events reviewed at interventional radiology morbidity and mortality conferences to assess whether procedure timing was associated with differences in adverse event severity and quality-of-care assessment.

**Results:**

A total of 547 adverse events occurring across four affiliated hospital sites were included. The cohort comprised 341 males and 206 females with a mean age of 56.2 years. Vascular procedures accounted for 369 events (67.5%), and non-vascular procedures accounted for 178 events (32.5%). Most adverse events occurred during weekday daytime hours (*n* = 459, 83.9%), followed by weekday after-hours (*n* = 50, 9.1%) and weekend periods (*n* = 38, 6.9%). Compared with weekday daytime procedures, a greater proportion of adverse events resulting in death occurred during weekday after-hours and on weekends, as reflected by a higher proportion of Society of Interventional Radiology class F events and Harm Score 9 outcomes (*p* < 0.001). In contrast, no significant difference in Quality Control System scores was observed across procedure timing categories (*p* = 0.73).

**Conclusions:**

Among adverse events reviewed at interventional radiology morbidity and mortality conferences, procedures performed during weekday after-hours and weekend periods were associated with greater adverse event severity, while quality-of-care assessments did not differ by procedure timing. These findings describe patterns within morbidity and mortality-reviewed complications and do not estimate complication rates or population-level risk by procedure timing. The absence of variation in Quality Control System scores supports the hypothesis that observed differences in severity may reflect patient acuity and procedural urgency rather than differences in procedural quality.

## Background

Interventional radiology (IR) plays a critical role in the management of both elective and urgent conditions, frequently providing minimally invasive therapies for patients with significant comorbidity and acute illness [1]. Despite advances in technique, technology, and peri-procedural care, adverse events continue to occur and remain an important focus of quality improvement and patient safety efforts within IR [2].

Across multiple procedural and surgical specialties, procedures performed during weekends or weekday after-hours have been associated with worse patient outcomes, including increased morbidity and mortality (M&M) [3]. Proposed contributors to these findings include differences in patient acuity, limited availability of ancillary resources, reduced staffing, and provider fatigue [3]. This phenomenon, often referred to as the “weekend effect”, has been described in vascular surgery, neurosurgery, spine surgery, and other high-acuity disciplines [4–7]. However, the extent to which procedure timing is associated with adverse event severity in IR remains unknown.

The purpose of this study was to retrospectively evaluate adverse events reviewed at IR M&M conferences over a ten-year period and to assess whether procedure timing (weekday daytime, weekday after-hours, or weekend) is associated with differences in adverse event severity and quality-of-care assessment.

## Methods

### Study design

The aim of this study was to evaluate whether procedure timing was associated with differences in adverse event severity and quality-of-care assessment among IR procedures. A retrospective observational study was performed using adverse events reviewed at the joint IR M&M conferences for four affiliated hospital sites within a single academic healthcare system. Institutional review board approval was obtained, and the study was conducted in compliance with the Health Insurance Portability and Accountability Act.

### Study population and case selection

Departmental IR M&M conference records were compiled and reviewed. A total of 641 adverse events were initially identified. Events were excluded if they represented duplicate entries (*n* = 67), occurred at non-affiliated institutions (*n* = 11), or lacked sufficient clinical information for analysis (*n* = 16), including cases in which no IR procedure was performed or the adverse event was deemed unrelated to the IR procedure. The final study cohort comprised 547 adverse events.

### Data collection and variable definitions

Electronic medical record and imaging review were performed to extract demographic and procedural information, including patient age, sex, hospital site, procedure type (vascular or non-vascular), and procedure start time. Procedure timing was categorized into three predefined groups based on start time: weekday daytime (Monday-Friday, 8:00 AM to 5:00 PM), weekday after-hours (Monday-Friday, 5:00 PM to 8:00 AM), and weekend (Saturday 8:00 AM through Monday 8:00 AM).

Each adverse event had been previously reviewed during monthly IR M&M conferences and assigned severity and quality classifications as part of routine departmental quality assurance. Adverse event severity was categorized using the Society of Interventional Radiology (SIR) complication classification system (Classes A-F) [8]. Patient outcome severity was additionally characterized using a Harm Score ranging from 1 (unsafe condition) to 9 (death). Quality-of-care assessment was evaluated using an internal Quality Control System (QCS) score, reflecting IR faculty consensus regarding the presence and severity of quality-of-care concerns associated with each adverse event. These classification systems are summarized in Tables 1, 2, and 3.

**Table 1:** QCS Classifications for Quality-of-Care Assessment

**QCS Classification**	**Description**
QCS 0	No quality-of-care concern identified
QCS 1	Near miss or event reached patient – Low risk
QCS 2	Near miss – High risk to patient
QCS 3	Event reached patient – Additional care required
QCS 4	Event reached patient – Potentially life-threatening or disabling
QCS 5	Event reached patient – Life-threatening or fatal

**Table 2:** Harm score definitions for patient outcome severity

Harm score	Description
1	Unsafe condition
2	Near miss – Did not reach the patient
3	Event reached patient – No harm evident
4	Emotional distress or inconvenience
5	Additional treatment required
6	Temporary harm – Bodily injury
7	Permanent harm – Minor lifelong effect
8	Severe permanent harm – Disabling lifelong effect
9	Death

**Table 3:** SIR Classification for Complications by Outcome [8]

Minor Complications	
A	No therapy, no consequence
B	Nominal therapy, no consequence; includes overnight admission for observation only
Major Complications	
C	Require therapy, minor hospitalization (< 48 h)
D	Require major therapy, unplanned increased in level of care, prolonged hospitalization (> 48 h)
E	Permanent adverse sequelae
F	Death

### Outcomes

The primary outcomes of interest were adverse event severity as measured by SIR classification and Harm Score, stratified by procedure timing category. A secondary outcome was the distribution of QCS scores across procedure timing categories to assess whether perceived quality-of-care concerns differed by timing.

### Statistical analysis

Descriptive statistics were used to summarize demographic, procedural, and outcome characteristics. Categorical variables were compared across procedure timing categories using chi-square testing. Continuous variables were assessed using one-way analysis of variance following evaluation for homogeneity of variance. Statistical analyses were performed using spreadsheet-based data analysis software (Excel 2019; Microsoft, Redmond, WA) and open-source statistical software (jamovi, Version 1.6; The jamovi project, Sydney, Australia). A *p*-value < 0.05 was considered statistically significant.

## Results

### Study cohort and demographics

A total of 547 adverse events reviewed at IR M&M conferences were included in the final analysis after exclusions. The cohort comprised 341 males and 206 females, with a mean age of 56.2 years. Adverse events occurred across four affiliated hospital sites, with the highest proportion observed at the primary tertiary care academic center. Procedures associated with adverse events were predominantly vascular (*n* = 369, 67.5%), with the remaining events occurring after non-vascular procedures (*n* = 178, 32.5%). Demographic and procedural characteristics stratified by hospital site are summarized in Table 4.

**Table 4:** Patient and procedural characteristics by hospital site

Characteristic	Total	Site 1	Site 2	Site 3	Site 4
Adverse Events	547	289	146	89	23
Male	341	154	94	84	9
Female	206	135	52	5	14
Mean age (years)	56.2	55.6	52.7	65.5	49.5
Non-vascular procedures	178	100	54	24	0
Vascular procedures	369	189	92	65	23

Values are presented as number of adverse events

### Distribution of adverse events by procedure timing

Most adverse events occurred during weekday daytime hours (*n* = 459, 83.9%), followed by weekday after-hours periods (*n* = 50, 9.1%) and weekends (*n* = 38, 6.9%). There was no significant difference in patient age across procedure timing categories (F(2, 544) = 0.20, *p* = 0.82). Patient demographics and procedure type stratified by procedure timing are summarized in Table 5.

**Table 5:** Patient and procedural characteristics by procedure timing

Characteristic	Weekday Daytime	Weekday After-hours	Weekend
Adverse Events	459	50	38
Male	288	32	21
Female	171	18	17
Mean age (years)	56.3	55.0	56.9
Non-vascular procedures	149	17	12
Vascular procedures	310	33	26

Values are presented as number of adverse events

### Adverse event severity and outcomes

Quality-of-care assessment, as measured by the QCS score, did not differ significantly across procedure timing categories (*p* = 0.73), as summarized in Table 6. In contrast, higher-severity patient outcomes, including Harm Score 9 (death), occurred more frequently during weekday after-hours and weekend procedures compared with weekday daytime procedures (*p* < 0.001). These results are summarized in Table 7. Similarly, the distribution of SIR complication classifications varied by procedure timing, with a greater proportion of SIR Class F events (death) observed during weekday after-hours and weekend periods compared with weekday daytime hours (*p* < 0.001), as shown in Table 8.

**Table 6:** Distribution of QCS scores by procedure timing

QCS score	Weekday Daytime	Weekday After-hours	Weekend
0	395	46	30
1	10	0	1
2	10	2	1
3	36	2	5
4	4	0	0
5	4	0	1

Values are presented as number of adverse events

**Table 7:** Distribution of harm scores by procedure timing

Harm Score	Weekday Daytime	Weekday After-hours	Weekend
0	1	1	0
1	15	1	2
2	4	0	0
3	59	10	9
4	19	3	0
5	247	14	14
6	54	3	0
7	5	0	1
8	2	1	0
9	53	17	12

Values are presented as number of adverse events

**Table 8:** Distribution of SIR adverse event classifications by procedure timing

SIR Classification	Weekday Daytime	Weekday After-hours	Weekend
A	63	11	9
B	62	3	2
C	180	9	7
D	91	9	7
E	10	1	1
F	53	17	12

Values are presented as number of adverse events

## Discussion

Adverse events are an important consideration in IR and remain a key focus of patient safety and quality assurance efforts in clinical practice. While some adverse events may relate to technical factors or procedural planning, others are influenced by patient acuity and the urgent nature of many interventions, particularly outside routine operating hours. In this retrospective review, procedures performed during weekday after-hours and weekend periods were associated with greater adverse event severity, while quality-of-care assessments did not differ by procedure timing. The predominance of adverse events during routine weekday hours at the primary academic center likely reflects higher procedural volume and referral complexity. Together, these findings are consistent with the hypothesis that differences in patient acuity and case urgency contribute to the observed variation in adverse event severity.

Prior studies across procedural specialties have described worse outcomes associated with weekend or after-hours care, commonly referred to as the “weekend effect”. These observations have been attributed to a combination of system-level and human factors, including inadequate staffing, resource availability, and operator fatigue during off-hours [3]. However, IR practice differs from many surgical disciplines in that after-hours and weekend procedures are typically restricted to urgent or emergent indications, while elective cases are scheduled during routine weekday hours [9]. As a result, IR patients undergoing procedures during these periods often represent a more critically ill population with higher baseline risk. Similar patterns have been described in prior studies across acute care settings, where weekend admissions are associated with greater patient illness severity and higher mortality risk, even after adjustment for standard clinical characteristics [10]. The present findings align with this literature and support the interpretation that observed differences in adverse event severity may reflect underlying case mix rather than differences in the quality of procedural care delivered.

The absence of a significant difference in QCS scores across procedure timing categories is a notable finding. QCS scoring reflects faculty consensus regarding potential quality-of-care concerns identified during M&M review, and the lack of variation across timing categories suggests that procedural decision-making and technical performance were not compromised outside the regular operating hours.

### Methodological considerations and bias

This analysis evaluates a captured cohort of adverse events reviewed through departmental M&M processes rather than a population-based sample of all IR procedures performed during the study period. Accordingly, the findings describe patterns among M&M-reviewed complications and do not estimate complication incidence, relative risk, or population-level differences in outcomes by procedure timing. Conditioning the analysis on the occurrence of an adverse event introduces important methodological considerations, including the potential for selection and collider bias. Indeed, conditioning on a variable that is influenced by multiple upstream factors can induce spurious associations between those factors, even in the absence of a direct causal relationship [11].

In the present study, the presence of an adverse event may be influenced by both procedure timing and patient acuity. By restricting the analysis to cases in which a complication occurred, the study conditions on this shared downstream outcome. This conditioning can distort observed relationships between timing and severity within the selected cohort. Such selection may artificially amplify apparent differences in adverse event severity across timing categories. For example, if after-hours and weekend procedures are predominantly high-acuity emergencies, while weekday daytime procedures encompass a broader range of acuity among cases presented at M&M, the subset of weekend complications may disproportionately represent critically ill patients. Under these circumstances, greater severity observed within the after-hours or weekend cohort may reflect underlying case mix rather than differences attributable to timing itself.

Addressing this potential bias would require additional data not available in the present study. Such data includes total procedural volumes stratified by timing to establish denominators, as well as objective measures of baseline patient acuity such as American Society of Anesthesiologists (ASA) scores, shock index, or other validated risk stratification variables. Incorporation of these parameters would allow evaluation of whether procedure timing independently contributes to adverse event risk or severity after adjustment for patient-level factors.

Other methodological limitations include the definition of procedure timing by start time, without separate analysis of cases extending across timing categories. Information regarding operator experience and duty hours was also not available, limiting assessment of potential human performance factors. In addition, as this retrospective analysis reflects adverse events reviewed through a single academic healthcare system’s quality assurance process, the findings may differ across institutions with different staffing models or case-mix distributions.

## Conclusions

In conclusion, this analysis describes patterns observed within M&M-reviewed complications and does not estimate population-level risk, adverse event incidence, or causal effects of procedure timing. Greater severity among after-hours and weekend complications, in the absence of differences in quality-of-care assessment, is consistent with differences in patient acuity and case urgency within the reviewed cohort. As the scope and volume of IR procedures continue to expand, continued evaluation of adverse event severity within the appropriate clinical context remains essential for informed quality improvement and institutional practice planning.

## Data Availability

All data generated or analysed during this study are included in this published article.

## Abbreviations

IR: Interventional Radiology
M&M: Morbidity and Mortality
SIR: Society of Interventional Radiology
QCS: Quality Control System
ASA: American Society of Anesthesiologists
